# The Different Effects of BMI and WC on Organ Damage in Patients from a Cardiac Rehabilitation Program after Acute Coronary Syndrome

**DOI:** 10.1155/2015/942695

**Published:** 2015-07-13

**Authors:** Lin Xu, Hui Zhao, Jian Qiu, Wei Zhu, Hongqiang Lei, Zekun Cai, Wan-Hua Lin, Wenhua Huang, Heye Zhang, Yuan-Ting Zhang

**Affiliations:** ^1^Department of Cardiology, Guangzhou General Hospital of Guangzhou Military Region, PLA, Guangzhou, Guangdong 510010, China; ^2^Guangzhou University of Chinese Medicine, Guangzhou 510403, China; ^3^Institute of Biomedical and Health Engineering, Shenzhen Institutes of Advanced Technology, Shenzhen 518055, China; ^4^Key Laboratory for Health Informatics, Chinese Academy of Sciences, Shenzhen 518055, China; ^5^Department of Anatomy, Guangdong Provincial Key Laboratory of Medical Biomechanics, School of Basic Medical Science, Southern Medical University, Guangzhou 510515, China; ^6^Joint Research Center for Biomedical Engineering, The Chinese University of Hong Kong, Shatin, New Territories 999077, Hong Kong

## Abstract

One of the purposes of cardiac rehabilitation (CR) after acute coronary syndrome (ACS) is to monitor and control weight of the patient. Our study is to compare the different obesity indexes, body mass index (BMI), and waist circumference (WC), through one well-designed CR program (CRP) with ACS in Guangzhou city of Guangdong Province, China, in order to identify different effects of BMI and WC on organ damage. In our work, sixty-one patients between October 2013 and January 2014 fulfilled our study. We collected the vital signs by medical records, the clinical variables of body-metabolic status by fasting blood test, and the organ damage variables by submaximal exercise treadmill test (ETT) and ultrasonic cardiogram (UCG) both on our inpatient and four-to-five weeks of outpatient part of CRP after ACS. We mainly used two-tailed Pearson's test and liner regression to evaluate the relationship of BMI/WC and organ damage. Our results confirmed that WC could be more accurate than BMI to evaluate the cardiac function through the changes of left ventricular structure on the CRP after ACS cases. It makes sense of early diagnosis, valid evaluation, and proper adjustment to ACS in CRP of the obesity individuals in the future.

## 1. Introduction

Obesity usually caused a variety of structural adaptations/alterations that could largely damage cardiovascular structure/function [1]. And cardiac rehabilitation (CR) which is public and well-developed has become a comprehensive management of cardiovascular disease (CVD) clinically [2, 3]. Therefore, one of the purposes of CR after acute coronary syndrome (ACS) is to monitor and control weight of the patient [4]. Body mass index (BMI) has been a well-accepted index to evaluate the overweight or obese individuals, but it has been reported that BMI fails to reflect true body composition in many studies [5–8]. Gruberg et al. have found the obesity paradox phenomenon, which is the fact that obese patients seemed to survive better in cardiovascular disease populations [9, 10]. One possible explanation of that is that the popular index of general obesity, BMI, might not be able to accurately reflect the distribution of body fat and fat free mass (FFM) [11–14]. Generally, the increase of body fat is easier to cause CVD than FFM because of metabolic abnormalities [1]. In contrast, waist circumference (WC), a common index to diagnose central obesity, can better reflect body fat than BMI. In other words, WC also has been associated with an increased risk of mortality in patients with CVD [15]; furthermore it might be significant to the prognosis of heart disease. de Koning et al. reported a study that consisted of 15,923 subjects with CVD and 5,696 deaths after a median follow-up of 2.3 years. It is said that one centimeter increased in WC was associated with 2% increase in men and 5% in women at the risk of future cardiovascular events [16]. Some recent studies explained that high WC could be a potential predictor of endothelial dysfunction, vascular damage and inflammation, and so forth [17–19]. Miyazaki et al. studied 98 patients at 1-2 weeks and at 6 months after ACS, and had founded that the decrease of WC showed to be more related to the progress of endothelial function than the decrease of BMI [17]. Lee et al. reported that it is the WC but not BMI that can reflect the remodeling process after anterior-wall acute myocardial infarction [20]. Moreover, another study also reported that the introduction of CR could have positive effect on BMI, WC, plasma lipoprotein status, and hypertension in obese patients [21]. However, little information is available on the different performance of the BMI and WC indices to associate on the prognosis indexes of CR after ACS, such as the organ damage like the change of cardiovascular structure/function, mortality, and morbidity.

For a better long-term prognosis, we need to monitor and control obesity effectively in CR after ACS. But, before that, we have to confirm the accurate relationship between the severity of obesity and the prognosis. Our study is to compare the different obesity indexes, BMI, and WC, through one well-designed CR program (CRP) on middle-aged and young patients with ACS, in order to identify the prognosis-value of WC in supervising and reducing obesity in the CRP after ACS, especially in the part of the observing organ damage.

## 2. Materials and Methods

### 2.1. Study Population

The study was conducted in the General Hospital of Guangzhou Military Command of People's Liberation Army (PLA), Guangdong Province, China. Sixty-one patients referred to CRP between October 2013 and January 2014 after percutaneous coronary intervention (PCI) or thrombolytic therapies were recruited into our study. Sixty-one individuals aged (49.7 ± 7.9) years (93.3% males gender) were enrolled in our study. The subjects in this study fulfilled the following inclusion criteria: (1) being aged 18–60 years, (2) established ACS, (3) being suitable for the CRP, (4) no weight reduction drug treatment or surgery before participating in this study, and (5) the existence of coronary artery lesions confirmed by coronary angiography (CAG) at the entry. However, the patients with severe complications were eliminated from this study because of potential inconvenience during the CRP, and these severe complications included malignant arrhythmia, acute congestive heart failure decomposition, severe acute pericarditis, myocarditis, systemic inflammatory, cachexy, serious chronic disease, recent thrombosis, or nervous musculoskeletal diseases.

### 2.2. Measurements

The eligible participant was first directed to finish the medical record that contains the medical information as follows: (1) demographics: age, gender, cardiovascular risk factors including smoking, hypertension, dyslipidemia, and diabetes, and so forth and (2) previous medical history of PCI or thrombolytic therapies, and so forth. Then, vital signs, which were heart rate (HR), blood pressure (BP), diastolic blood pressure (DBP), and weight, were measured after the subject was asked to sit quietly for five minutes. Blood pressure was measured on the left arm. Sixty-one subjects completed this measurement.

#### 2.2.1. The Measurement of BMI, WC

BMI and WC are two measures of obesity in this study. BMI was calculated as weight (in kilograms) divided by the square of height (in meters), and WC was assessed at the midpoint, the narrowest point between the lowest rib and the iliac crest, to the nearest 0.1 cm using an inflexible tape measure.

We classified the patients into normal group and abnormal group on the basis of BMI and WC, respectively. Abnormal BMI and WC were defined according to the NIH Practical Guide to Obesity and the International Diabetes Federation (IDF) guidelines which were informed by the World Health Organization (WHO) in 2008 [22]. Patients who satisfied the criteria of either overweight or obesity were classified as abnormal group. Specifically, patients with BMI ≥ 25 kg/m^2^ were identified as abnormal, and males with WC > 90 cm or females with WC > 80 cm were stratified as abnormal [22]. Sixty-one subjects completed this measurement.

#### 2.2.2. Fasting Blood Test

Routine fasting blood test was carried out in the department of clinical biochemistry to collect the blood parameters of fasting blood glucose (FBG, in mmol/L), serum uric acid values (UA, in umol/L), and serum lipids values including total cholesterol (TC, in mg/dL), triglycerides (TG, in mg/dL), low density lipoprotein cholesterol (LDL_C, in mg/dL), and high density lipoprotein cholesterol (HDL_C, in mg/dL). Generally, FBG, serum lipids and UA values represent the metabolic status of our body. The metabolic abnormalities can induce atherosclerosis, thus leading to systematic organ damage [23]. Sixty-one subjects completed this measurement.

#### 2.2.3. Submaximal Exercise Treadmill Test (ETT)

Submaximal ETT was carried out according to the Bruce protocol using a sports test machine (CASE, GE Medical Systems: Critikon, Chihuahua). Because of some restrained conditions including the psychological factors and unsuitable symptom, only 21 cases completed the ETT. We documented the parameters as follows: treadmill exercise time (in min), metabolic equivalents (METs), maximum net ST segment deviation (depression or elevation), and Duke treadmill score (DTS). Among them, treadmill exercise time and METs reflect the exercise performance directly and represent the cardiopulmonary function indirectly. Maximum net ST segment deviation and DTS may predict suspected cardiovascular disease (CVD). Twenty-one subjects completed this measurement.

#### 2.2.4. Ultrasonic Cardiogram (UCG)

UCG was carried out in the department of echocardiography with an ultrasonic electrocardiogram machine (IE33 S/N, Philips Medical Systems, US). We documented the parameters as follows: left ventricular end diastolic dimension (LVDd, mm), interventricular septal thickness at diastole (IVSd, mm), left ventricular posterior wall at diastole (LVPWd, mm), ejection fraction (EF, %), and fractional shortening (FS, %). Among them, LVDd, IVSd, and LVPWd were three important indices that can reflect the cardiac structure of left ventricular (LV), and EF and FS were two important indices that can reflect the cardiac ejection function. The enlargement of LV structure and the decline of cardiac ejection function reveal heart lesions. Sixty-one subjects completed this measurement.

#### 2.2.5. Coronary Angiogram (CAG)

CAG was performed to detect the lesions of the coronary vascular with a digital subtraction angiography machine (Allura Xper FD20, Philips Medical Systems Nederland B.V.). Left main coronary disease (LMD), three-vessel disease (TVD), and complicated coronary artery disease (CCAD) were diagnosed by doctors based on the results of CAG. PCI was implemented according to the guidance of doctor if the existence of coronary artery lesions were confirmed by the CAG. Sixty-one subjects completed this measurement.

#### 2.2.6. The Procedure of CRP

We designed a specific CRP for the patients participated in this study after the measurements above-mentioned. It consisted of two parts: an inpatient-phase part for two-to-three weeks followed by an early outpatient-phase part for four-to-five weeks. The inpatient-phase part was for education and counseling of the information of the CRP, risk factor management, nutrition and diet guidance, postoperative activity implementation to the patient, and psychological support, and so forth. The patients were also directed to do appropriate limb movements on a bed and simple walk training for getting out of bed as soon as possible, which could help them to get ready for the early outpatient-phase part. The early outpatient-phase part consisted of a five-minute warm-up and light exercise (stretching), a twenty-minute aerobic exercise (walking or trotting), and a ten-minute cooling-down period (stretching). Each patient carried out the training program three times per week. The exercise intensity was prescribed individually according to 60% of the maximum intensity at ordinary times or the patient's heart rate (HR) reached approximately 60% of the maximum HR (HR_max⁡_) calculated after ETT. During the training time, the subjective Brog score of patients was about 11-12 points.

#### 2.2.7. Follow-Up

Patients came back to visit us after CRP for four-to-five weeks. Medical records collection, fasting blood test, submaximal ETT, and UCG were performed again according to the measurements above-mentioned. In this period, sixty-one subjects completed the medical records and fasting blood test, thirty-seven subjects completed the ETT, and forty subjects completed the UCG test.

### 2.3. Statistical Analysis

First of all, baseline clinical characteristics and vital clinical variables of the subjects were studied for normal group and abnormal group, respectively. Categorical variables were presented as percentages, while continuous variables were present as means ± SD. The heterogeneity between normal group and abnormal group was assessed by independent-sample *t* tests and chi-square test. Independent-sample *t* test was used for the analysis of continuous variables, while chi-square test was for categorical variables. *P* value < 0.05 was considered to be significant. Secondly, the correlation of different measures of obesity (i.e., BMI and WC) with vital clinical variables was analyzed using two-tailed Pearson's test. The vital clinical variables in this study included blood pressure, the values of body-metabolic status (i.e., FBG, TC, TG, LDL_C, HDL_C, and UA), variables for the measurement of cardiac structure (i.e., LVDd, IVSd, and LVPWd), and variables for the measurement of cardiac function (i.e., EF, FS, treadmill exercise time, METs, maximum net ST segment deviation, and DTS). Furthermore, a simple linear regression analysis was applied to detect the linear relationship between the measures of obesity and the cardiac structure alteration (i.e., IVSd and LVPWd). After that, multiple linear regression analysis using the backward selection method was performed to estimate the effects of measures of obesity on cardiac structure alteration. WC and BMI were used as independent factors. *P* value of 0.1 was the criterion for a variable to remain in the model, and *P* value less than 0.05 was considered statistically significant. Finally, the multiple linear regression analysis was adjusted by age, smoking, hypertension, diabetes, TC, and HDL_C, which were the risk factors used for predicting coronary heart disease in the Framingham Risk Score [24], to verify which measure of obesity was independent of the traditional cardiovascular risk factors. All the analyses were carried out before the CRP and during the follow-up period after CRP twice. The statistical analysis was carried out using SPSS (IBM Company, USA).

## 3. Results

Table 1 shows the baseline clinical characteristics of the sixty-one subjects. Mean ± SD age was 49.7 ± 7.9 years, and 93.3% were men. Based on BMI standard, 34 and 27 cases were categorized into normal group and abnormal group, respectively. However, based on WC standard, 21 cases and 40 cases were categorized into normal group and abnormal group, respectively. The percentage of subjects with the risk factor of hypertension was significantly higher in the abnormal group than in the normal group regardless of being stratified by BMI or WC standard. The values were 70.4% versus 26.5% (*P* < 0.01) and 57.5% versus 23.8% (*P* < 0.05) for the two groups that were stratified by BMI and WC standard, respectively. And, there were no significant differences of age, gender, smoking, diabetes, dyslipidemia, left main disease (LMD), three-vessel disease (TVD), and complicated coronary artery disease (CCAD) in the two groups regardless of being stratified by BMI or WC standard.

Table 2 shows the vital clinical variables of the subjects of normal group and abnormal group stratified by BMI and WC standard before CRP. Subjects with abnormal WC had significantly higher SBP than those with normal WC (128.5 ± 21.9 mmHg versus 120.6 ± 13.3, *P* < 0.05). However, the difference was not significant between the two groups stratified by BMI standard. Beyond that, other vital variables examined by fast blood test, UCG, and ETT appeared as no significant differences between the normal group and the abnormal group regardless of being stratified by BMI or WC standard.

Table 3 depicts the vital clinical variables of the subjects of normal group and abnormal group stratified by BMI and WC standard after CRP. They appeared as several differences in subjects stratified by BMI and WC standard. Firstly, based on BMI standard, 32 and 29 subjects were categorized into normal group and abnormal group, respectively. However, based on WC standard, 24 subjects and 37 subjects were categorized into normal group and abnormal group, respectively. Secondly, subjects with abnormal BMI possessed significantly higher DBP, longer average treadmill exercise time than those with normal BMI. The values were 75.7 ± 9.6 mmHg versus 68.4 ± 7.7 mmHg (*P* < 0.01) and 8.9 ± 2.1 min versus 7.5 ± 1.6 min (*P* < 0.05), respectively. However, these differences were not significant between the two groups stratified by WC standard. Thirdly, subjects with abnormal WC possess significantly lower HDL_C, higher IVSd, and higher LVPWd than those with normal WC. The values were 41.2 ± 9.5 mg/dL versus 47.6 ± 15.3 mg/dL (*P* < 0.05), 9.7 ± 1.6 mm versus 8.4 ± 1.1 (*P* < 0.01), and 12.3 ± 2.3 mm versus 11.0 ± 1.3 mm (*P* < 0.05), respectively. However, these differences were not significant between the two groups stratified by BMI standard.

**Figure 1:**
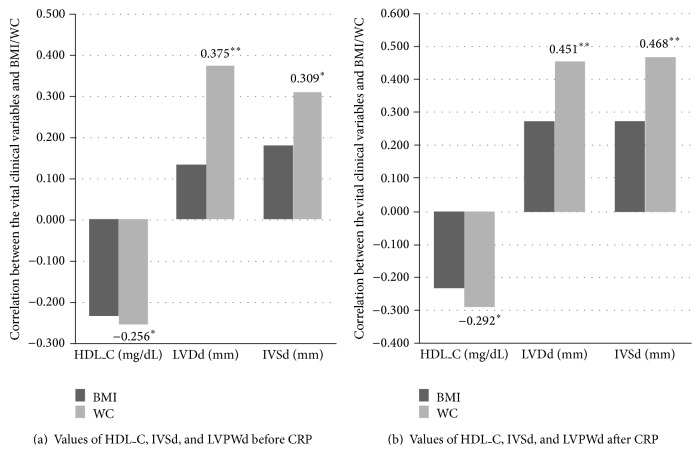
The correlation of the significant variables and BMI/WC in all subjects before and after CRP.* Notes*: ^*^correlation is significant at the 0.05 level. ^**^Correlation is significant at the 0.01 level. CRP: cardiac rehabilitation program; BMI: body mass index; WC: waist circumference; SBP: systolic blood pressure; DBP: diastolic blood pressure; IVSd: interventricular septal thickness at diastole; LVPWd: left ventricular posterior wall at diastole.

Table 4 depicts Pearson's correlation coefficients of vital clinical variables above-mentioned to BMI and WC before and after CRP. As shown in the table, firstly, BP index (both SBP and DBP) appeared as a positive correlation with BMI both before and after the CRP (SBP before CRP: *r* = 0.287, *P* = 0.025; SBP after CRP: *r* = 0.461, *P* = 0.000; DBP before CRP: *r* = 0.279, *P* = 0.029; DBP after CRP: *r* = 0.542, *P* = 0.000), while the correlation was only significant for WC after CRP (SBP after CRP: *r* = 0.371, *P* = 0.003; DBP after CRP: *r* = 0.309, *P* = 0.015). Secondly, the IVSd and LVPWd showed a positive correlation while the HDL_C appeared as a negative correlation with WC but not BMI before CRP and after CRP (IVSd before CRP: *r* = 0.375, *P* = 0.003; LVPWd before CRP: *r* = 0.309, *P* = 0.016; HDL_C before CRP: *r* = −0.256, *P* = 0.046. IVSd after CRP: *r* = 0.451, *P* = 0.004; LVPWd after CRP: *r* = 0.468, *P* = 0.002; HDL_C after CRP: *r* = −0.292, *P* = 0.022). Besides, the FBG, UA, and LDL_C showed a positive correlation with WC but not BMI before CRP (FBG: *r* = 0.305, *P* = 0.017; UA: *r* = 0.369, *P* = 0.003; LDL_C: *r* = 0.334, *P* = 0.009).  Figure 1 shows the different correlation coefficients of BMI and WC to HDL_C, IVSd, and LVPWd before and after CRP.

**Figure 2:**
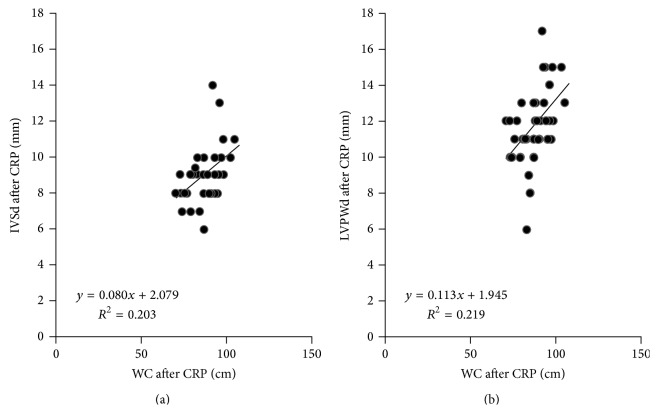
Scatter plots showing the linear correlation between IVSd/LVPWd and WC after CRP. CRP: cardiac rehabilitation program; BMI: body mass index; WC: waist circumference; IVSd: interventricular septal thickness at diastole; LVPWd: left ventricular posterior wall at diastole.

Table 5 shows the simple linear regression outcomes after CRP. The results showed that WC had a highly linear correlation with indices that reflected cardiac structure alteration (LVSd after CRP: *R* ^2^ = 0.203, *P* = 0.004; LVPWd after CRP: *R* ^2^ = 0.219, *P* = 0.002). The correlation scatter diagram was shown in Figure 2. However, BMI had no linear relationship with LVSd and LVPWd (*P* > 0.05).

Table 6 shows the outcomes of multiple linear regression analysis after CRP. In Model 1, LVSd is dependent factor, while WC and BMI as independent factors were entered into the model. The results showed that WC was the only significant factor (adjusted *R* ^2^ = 0.182, *P* = 0.004) remaining in the model. After adjusted by age, smoking, hypertension, diabetes, TC, and HDL_C, WC still kept in significant correlation with LVSd (Model 2, adjusted *R* ^2^ = 0.305, *P* = 0.001). In Model 3, LVPWd is dependent factor, while WC and BMI as independent factors were entered into the model. The results showed that WC was the only significant factor (adjusted *R* ^2^ = 0.198, *P* = 0.002) remaining in the model. After adjusted by age, smoking, hypertension, diabetes, TC, and HDL_C, WC still kept in significant correlation with LVPWd (Model 4, adjusted *R* ^2^ = 0.269, *P* = 0.002).

## 4. Discussions

This study estimated the different effects of BMI and WC on organ damage in subjects from a CRP after ACS. There were five key findings: (1) obesity assessed by increasing WC was significantly associated with lower HDL_C, higher LVSd, and higher LVPWd in patients from a CRP after ACS. However, the associations were insignificant when the obesity was assessed by BMI. (2) WC had a highly linear correlation with indices that reflected cardiac structure alteration while BMI is not. (3) WC was the only significant factor remaining in the model when a multiple linear regression analysis was performed to estimate the effects of WC and BMI on cardiac structure alteration. (4) After adjusted by age, smoking, hypertension, diabetes, TC, and HDL_C, WC still kept in significant correlation with cardiac structure alteration. (5) Obesity assessed by increasing BMI was significantly associated with higher SBP and DBP in patients from a CRP after ACS. However, when the obesity was assessed by WC, the associations were significant only for the subjects after CRP but not for the subjects before CRP.

### 4.1. Overweight/Obesity Individuals Assessed by WC and BMI Appeared Different Significant Vital Clinical Variables

Our study shows that the different standard indexes on predicting the degree of obesity may induce the discrepancies of the population characteristics.

Before CRP, the prevalence of hypertension on abnormal BMI/abnormal WC patients is higher than the normal BMI/normal WC patients (Table 1), but *P* value of the BMI group is larger than WC. And the abnormal WC patients got higher SBP than normal WC patients (Table 2). After CRP, the abnormal BMI patients got higher DBP and shorter treadmill exercise time than normal BMI patients: however, the abnormal WC patients had undergone the lower HDL_C and higher IVSd/LVPWd values than normal WC patients (Tables 2 and 3). Thus, the results show that overweight/obesity individuals assessed by WC and BMI appeared as different vital clinical variables characteristics. Moreover, regardless of being grouped by BMI or WC, overweight/obesity individuals appear as worse parameter of prognosis than the nonobese individuals. That is mainly because the chronic accumulation of the body fat would lead to the bunch of pathophysiological changes such as the increase of cardiac output, inflammation, metabolic abnormalities, and the atherosclerosis [1]. Such pathophysiological changes play an important role in the organ damage. That is to say, it is consistent with the results of previous researches that the development of organ damage exists in the overweight/obesity individuals.

### 4.2. Correlation of WC with Cardiac Structure in CRP after ACS

In this study, the finding that obesity assessed by increasing WC was significantly associated with lower HDL_C, higher LVSd, and higher LVPWd in patients from a CRP after ACS mirrors the results of previous researches [8, 25]. It has proved that overweight and obesity have many adverse effects on hemodynamic and cardiovascular structure and function [8]. The obesity often leads to the increases of filling pressure and volume in the heart, which would increase cardiovascular work, and leads to LV changes with dilation and LV hypertrophy [25]. As we have known, measures of cardiac structure, such as LVDd, IVSd, and LVPWd, are the most important parameters to early evaluate the size of LV structure which could reflect the cardiac function [26]. It is significant to observe the variation of LV structure in the CRP after ACS, especially on overweight/obesity individuals. BMI and WC are the parameters of obesity, but their functions on evaluating obesity are different to some extent. So the discrepancy between the BMI and WC indexes on the association of the organ damage also existed.

In our study, no matter before and after the CRP, the IVSd and LVPWd showed a positive correlation while the HDL_C showed a negative correlation with WC. However, BMI could not reflect this phenomenon. Furthermore, linear regression analyses told us that not only the simple but also the multiple linear relationship of WC to IVSd and LVPWd showed the statistically significant correlations (Tables 5, 6 and Figure 2). While WC and BMI especially entered in the same models, only WC was the only significant factor to associate with the IVSd and LVPWd. Because the larger values of IVSd and LVPWd represent the more serious compensatory hypertrophy changes of LV structure. We can draw a conclusion that WC is closer associated with the IVSd, LVPWd indexes which represent the cardiac structure than BMI in the CRP after ACS.

A potential mechanism to explain such association is that WC represents central obesity, which can exactly predict the body fat [1]. The increased body fat are easier to cause CVD [27], because of the adipose tissue induce the progress of metabolic disturbance, inflammation state, the neuroendocrine changes, and atherosclerosis including the peripheral vessel and coronary artery, and so forth [28]. Moreover, central obesity will influence the cardiac structure and function through the increased cardiac load caused by hemodynamic disorder and the hypoxia or ischemia on the cardiovascular system [29–31]. All the changes of the cardiovascular system above-mentioned also lead to hypertension, arrhythmia, heart failure, and so forth [29–31]. They mostly reflected the compensatory hypertrophy changes of LV structure (LV remodeling) induced by the increased extra load on the cardiovascular system [29–31]. Furthermore, the results of some studies are consistent with our study. For examples, Turkbey et al. reported a study performed in an obese cohort of 5,098 participants (multiethnic study of atherosclerosis) and showed that higher LV mass-to-volume ratio changing in cardiac structure was linearly correlated with higher adiposity measurements such as the WHR, WC [32]. And Apridonidze et al. reported that increased waist circumference can strongly predict the LV hypertrophy [33]. According to results and analyzes above, WC should favorably be correlated with the cardiac structure on the CRP after ACS.

However, BMI is not significantly associated with the cardiac structure in our study. That is probably because BMI is easy to be affected by the weight of bone and muscle, which cannot truly reflect the body fat [34]. Besides, BMI fails to represent the individual difference about the distribution area of adipose tissue of our body [35]. In other words, BMI cannot distinguish the increased mass of fat and lean, so its information on general obesity could be suspicious. There is limited research on comparing BMI and WC to be associated with cardiac structure directly. Yet, Canepa et al. reported a study that WC and BMI were both associated with LV diastolic dysfunction when they were included in the logistic regression models separately. However, when both WC and BMI were in the same model, only WC remained significantly associated with LV diastolic dysfunction [36]. Bombelli et al. reported that, comparing to BMI, WC was the only index that showed statistically significant association with the trend of LV mass index after 10-year follow-up [37]. That is to say, the correlation of BMI and the cardiac structure or function on the CRP after ACS may not be accurate.

Moreover, the results of our study also indicated that the FBG, UA, and LDL_C indexes presented a positive correlation with WC just before CRP and the HR index showed a positive correlation with WC after CRP, but BMI did not show the correlation before and after CRP (Table 5). The previous researches told us that obesity, hypertension, dyslipidemia, insulin resistance and type 2 diabetes mellitus, and so forth are associated with the prevalence of CVD [1]; they are defined as cardiovascular risk factors. That is to say, WC may be significant to the correlation of the CVD, because of the more meaningful associations of vital clinical indexes than BMI.

Finally, the multiple linear relationship analysis also told us that age and diabetes together with WC have significant correlation with IVSd and the same as age and hypertension, together with WC to LVPWd on the CRP after ACS. In other words, age, hypertensions, and diabetes together with WC also make sense for the cardiac structure on CPR after ACS [38, 39]. As we know above, hypertension and diabetes are the cardiovascular risk factors and they must be relevant to the CVD and have the correlation with the cardiac function and structure. Besides, aging can cause the decline of the whole body function including the cardiovascular system. That might explain the results above.

### 4.3. BMI to Correlate with BP in CRP after ACS

According to the BMI standard group on our study, BMI is associated with BP both before and after CRP, and the positive correlation coefficient had mildly improved after CRP no matter SBP or DBP; the WC standard group also showed us the same positive correlation coefficient after CRP, the BMI standard group manifested more significantly different than WC (Table 5).

As we have known, obesity is associated with hypertension and the mechanisms are multifactorial [40]. At first, obesity can easily lead to dyslipidemia, insulin resistance and hyperinsulinemia, inflammation reaction [40]. Secondly, obesity could induce the endothelial dysfunction and increased vascular stiffness [28]. Thirdly, obesity also leads to the increased cardiac output, heart rate, and so forth which would increase the hemodynamic load to cause hypertension [40]. Fourthly, obesity can induce abnormal kidney function, such as the increased glomerular filtration rate (GFR), primary sodium retention, and activation of the renin-angiotensin system (RAS), and so forth [41]. In conclusion, the mechanisms above-mentioned totally could build up to the progress of atherosclerosis, which can contribute to hypertension. So the obesity indexes such as BMI/WC may be the significant correlation to BP on the CVD, especially in CRP after ACS. However, BM and WC also appear as different function in the correlation.

The result above-mentioned showed us that BMI could be more strongly associated with BP than WC. Some previous studies were consistent with these results. Oda and Kawai reported a local research and concluded that BMI, but not WC, was independently associated with hypertension in apparently healthy Japanese men and women [42]. Feng et al. also reported a study that BMI is strongly associated with hypertension and WC is strongly associated with diabetes [43]. Blumenthal et al. provided a study and concluded that for overweight or obese persons defined by BMI with above normal BP, exercise and weight loss could produce the larger BP reductions [44]. Mainly reasons are concluded as follows: (1) the Asia population have the larger prevalence on hypertension, when in the same BMI of different ethnic groups; (2) the increased BMI has more directly influence on the body fluid, the vascular resistance, and the heart output which can cause hypertension.

There also some diverse results on different studies, especially in the different gender or ethnic group. Sarno and Monteiro reported a study and concluded that both BMI and abdominal WC were positively and independently associated with the occurrence of arterial hypertension, but the influence of BMI was higher among men [45]. Warren et al. reported a study that abnormal WC was independently associated with a 5-fold risk in hypertension and diabetes in African American women [46]. The explanation of the contradiction is probably due to the different distribution of body fat and FFM between the different gender and ethnicity. Generally, WC which represented central obesity can better reflect body fat than BMI. Furthermore, body fat released lipids into the liver and secreted inflammatory cytokines, which can induce CVD [43, 47]. So we could infer that WC may also be associated with hypertension.

However, our study presented that BMI is more strongly associated with hypertension than WC and the reasons might be as follows: (1) the gender gap existed in our study (93.3% males gender), and the population characteristics also showed that overweight/obesity individual assessed by BMI is more statistically significant with hypertension than WC; (2) it was restricted on the outcomes to use the single BP measurement, and it will be more accurate to use the ambulatory BP measurement to record the BP variability; (3) the limitation of our sample size difference might also cause the outcomes; (4) different studies completed by different ethnic groups might be also considered. In other words, BMI can be associated with BP on the CRP after ACS, but we could not deny the association between WC and BP just judging the result from our study; it needs further studies to confirm their correlation.

### 4.4. BMI/WC Measurement in Clinical

As we know, WC represents the central obesity in clinical because it can reflect body fat [11–15]. Certain studies have shown that central obesity had a greater risk for CVD than BMI alone [48, 49]. Coutinho et al. reported on a systematic review of the literature and collaborative analysis from about 2,188 studies, suggesting that WC and waist-to-hip ratio (WHR) were more reliable than BMI in stratifying mortality risk in CVD patients [50]. There are also some studies showing a direct association of WC but not BMI with an increased risk for total mortality [15, 51]. It means that, to compare with BMI, WC may be more beneficial to evaluate the correlation of organ damage in CVD. It might also be the same performance of the CRP after ACS.

As our study shows the findings above, we can conclude that, to compare with BMI, WC could be more exactly relevant to evaluate the correlation of organ damage on CRP after ACS, especially in the change of LV structure. In other words, we can diagnose that the larger WC patient will get a worse consequence of suffering the heart failure or cardiomegaly and may also be a terrible situation of atherosclerosis progress. That is to say, besides BMI, WC measurement should be considered to focus on our clinical. For example, WC can be applied to evaluate the degree of obesity for the ACS patients, especially the ones in the CRP. It makes sense of early diagnosis, precise evaluation, and proper treatment to obesity individuals in CRP after ACS in the future clinical care.

### 4.5. Study Limitations

Nevertheless, our study also manifested shortcomings as follows. First of all, there are 61 cases in our study. The sample size is small, but we follow up all of them about 1 month after CRP. We also compare the data before and after CRP through observing the different effects of WC, BMI on organ damage evaluated by different indexes. Moreover, we also use different and proper statistical analysis methods such as the correlation analysis, simple and multiple linear correlation analysis. We conclude that comparing the BMI measurement, WC makes more sense for the evaluation of organ damage. Secondly, we did not analyze other obesity indexes such as WHR. Thirdly, we did not have the blank control group. Fourthly, we did not consider the influence of medication, diet, and PCI, and so forth. And then, there were not all patients had completed the UCG, EET. Furthermore, we did not follow up a long-term CRP to make more effective evidence. To make up with it, we will try more improved methods in the future.

## 5. Conclusions

Obesity is a risk factor for the development of cardiovascular events. CR is a rewarding progress to intervene in multiple cardiovascular risk profiles [4]. So if we can control obesity effectively via CRP, it means that we can reduce the cardiovascular events. In other words, to understand the accurate severity of obesity will make sense to evaluate the correlation of organ damage on CRP after ACS. Our study was about the analysis of the different effects of BMI, WC on the clinical indices including the vital signs, the clinical variables of body-metabolic status, and the organ damage variables indexes on our designed CRP after ACS. Our result confirmed that WC could be more exactly than BMI to evaluate the cardiac function through the changes of LV structure on the CRP after ACS cases. It makes sense of early diagnosis, valid evaluation, and proper adjustment to ACS in CRP of the obesity individuals in the future.

## Figures and Tables

**Table 1:** Clinical characteristics of the study subjects.

Characteristics	All subjects (*n* = 61)	BMI standard		WC standard	
		Normal (*n* = 34)	Abnormal (*n* = 27)	Normal (*n* = 21)	Abnormal (*n* = 40)
Age (years)	49.7 ± 7.9	50.4 ± 7.7	48.8 ± 8.1	49.5 ± 9.2	50.0 ± 7.2
Male gender (%)	93.3%	88.2%	96.3%	100.0%	87.50%
Smoking (%)	75.4%	73.5%	77.8%	76.2%	75.0%
Weight (kg)	69.8 ± 8.9	**64.7 ± 5.8** ^**^	**76.3 ± 7.8** ^**^	**64.6 ± 6.2** ^**^	**72.6 ± 8.9** ^**^
BMI (kg/m^2^)	24.9 ± 2.6	**23.1 ± 1.4** ^**^	**27.2 ± 1.9** ^**^	**23.4 ± 2.1** ^**^	**25.7 ± 2.5** ^**^
WC (cm)	91.9 ± 9.6	**89.2 ± 9.3** ^**^	**95.4 ± 8.9** ^**^	**82.4 ± 5.9** ^**^	**96.0 ± 7.0** ^**^
Diabetes (%)	36.1%	38.2%	33.3%	23.8%	42.5%
Hypertension (%)	45.9%	**26.5%** ^**^	**70.4%** ^**^	**23.8%** ^*^	**57.5%** ^*^
Dyslipidemia (%)	82%	82.4%	81.5%	71.4%	87.5%
LMD (%)	8.2%	5.9%	11.1%	0.0%	12.5%
TVD (%)	63.9%	61.8%	66.7%	52.4%	70.0%
CCAD (%)	73.8%	76.5%	70.4%	66.7%	77.5%

Notes: ^*^significance of difference is at the 0.05 level. ^**^Significance of difference is at the 0.01 level.

CRP: cardiac rehabilitation program; BMI: body mass index; WC: waist circumference; LMD: left main disease; TVD: three-vessel disease; CCAD: complicated coronary artery disease.

**Table 2:** Vital clinical variables of subjects of the normal group and abnormal group stratified by BMI and WC standards before CRP.

Variables	All subjects	BMI standard		WC classified	
		Normal	Abnormal	Normal	Abnormal
Smoking (%)	75.4% (*n* = 61)	73.5% (*n* = 34)	77.8% (*n* = 27)	76.2% (*n* = 21)	75.0% (*n* = 40)
HR (bpm)	83.2 ± 15.5 (*n* = 61)	83.2 ± 17.4 (*n* = 34)	83.3 ± 13.0 (*n* = 27)	82.4 ± 17.9 (*n* = 21)	83.6 ± 14.2 (*n* = 40)
SBP (mmHg)	128.5 ± 21.9 (*n* = 61)	126.7 ± 25.1 (*n* = 34)	130.8 ± 17.1 (*n* = 27)	**120.6** ± **13.3** ^*^ (*n* = 21)	**128.6** ± **13.6** ^*^ (*n* = 40)
DBP (mmHg)	74.5 ± 12.8 (*n* = 61)	72.7 ± 12.8 (*n* = 34)	76.8 ± 12.6 (*n* = 27)	72.3 ± 11.5 (*n* = 21)	75.6 ± 13.4 (*n* = 40)
FBG (mmol/L)	7.0 ± 3.0 (*n* = 61)	6.8 ± 2.5 (*n* = 34)	7.2 ± 3.5 (*n* = 27)	6.1 ± 1.9 (*n* = 21)	7.4 ± 3.4 (*n* = 40)
TC (mg/dL)	206.1 ± 62.2 (*n* = 61)	204.2 ± 59.2 (*n* = 34)	208.4 ± 66.9 (*n* = 27)	193.6 ± 47.3 (*n* = 21)	212.6 ± 68.4 (*n* = 40)
TG (mg/dL)	108.7 ± 147.1 (*n* = 61)	92.2 ± 140.3 (*n* = 34)	129.6 ± 155.2 (*n* = 27)	108.1 ± 166.7 (*n* = 21)	109.1 ± 137.9 (*n* = 40)
LDL_C (mg/dL)	123.6 ± 47.4 (*n* = 61)	119.9 ± 47.7 (*n* = 34)	128.3 ± 47.5 (*n* = 27)	110.2 ± 36.7 (*n* = 21)	130.7 ± 51.1 (*n* = 40)
HDL_C (mg/dL)	47.4 ± 13.5 (*n* = 61)	49.1 ± 14.4 (*n* = 34)	45.3 ± 12.3 (*n* = 27)	51.8 ± 16.3 (*n* = 21)	45.1 ± 11.4 (*n* = 40)
UA (umol/L)	368.7 ± 108.5 (*n* = 61)	366.7 ± 107.5 (*n* = 34)	371.1 ± 111.8 (*n* = 27)	337.1 ± 108.5 (*n* = 21)	385.3 ± 106.1 (*n* = 40)
LVDd (mm)	51.1 ± 5.5 (*n* = 61)	51.4 ± 5.3 (*n* = 34)	50.6 ± 5.8 (*n* = 27)	50.9 ± 6.8 (*n* = 21)	51.1 ± 4.7 (*n* = 40)
IVSd (mm)	9.2 ± 1.6 (*n* = 61)	9.1 ± 1.7 (*n* = 34)	9.3 ± 1.5 (*n* = 27)	8.8 ± 1.3 (*n* = 21)	9.4 ± 1.7 (*n* = 40)
LVPWd (mm)	11.7 ± 1.5 (*n* = 61)	11.6 ± 1.6 (*n* = 34)	11.8 ± 1.5 (*n* = 27)	11.3 ± 1.5 (*n* = 21)	11.9 ± 1.5 (*n* = 40)
EF (%)	58.7 ± 9.7 (*n* = 61)	57.0 ± 11.0 (*n* = 34)	60.7 ± 7.7 (*n* = 27)	57.7 ± 10.7 (*n* = 21)	59.2 ± 9.3 (*n* = 40)
FS (%)	31.8 ± 8.7 (*n* = 61)	31.0 ± 10.6 (*n* = 34)	32.7 ± 5.3 (*n* = 27)	32.0 ± 12.2 (*n* = 21)	31.7 ± 6.3 (*n* = 40)
Treadmill exercise time (min)	7.7 ± 1.7 (*n* = 21)	7.7 ± 1.7 (*n* = 11)	7.6 ± 1.8 (*n* = 10)	7.9 ± 1.9 (*n* = 9)	7.5 ± 1.6 (*n* = 12)
Metabolic equivalents	9.4 ± 2.4 (*n* = 21)	9.8 ± 2.8 (*n* = 11)	8.9 ± 1.8 (*n* = 10)	10.0 ± 3.2 (*n* = 9)	8.9 ± 1.6 (*n* = 12)
Maximum net ST segment deviation (mm)	1.1 ± 1.1 (*n* = 21)	1.2 ± 1.1 (*n* = 11)	1.1 ± 1.2 (*n* = 10)	1.3 ± 1.5 (*n* = 9)	1.0 ± 0.8 (*n* = 12)
DTS	0.6 ± 8.2 (*n* = 21)	0.8 ± 8.5 (*n* = 11)	0.3 ± 8.4 (*n* = 10)	1.4 ± 8.3 (*n* = 9)	0.0 ± 8.5 (*n* = 12)

Notes: ^*^significance of difference is at the 0.05 level. ^**^Significance of difference is at the 0.01 level.

CRP: cardiac rehabilitation program; BMI: body mass index; WC: waist circumference; HR: heart rate; SBP: systolic blood pressure; DBP: diastolic blood pressure; FBG: fasting blood glucose; LDL_C: low density lipoprotein cholesterol; TC: total cholesterol; TG: triglycerides; HDL_C: high density lipoprotein cholesterol; UA: serum uric acid; LVDd: left ventricular and diastolic dimension; IVSd: interventricular septal thickness at diastole; LVPWd: left ventricular posterior wall at diastole; EF: ejection fraction; FS: fractional shortening; DTS: Duke treadmill score.

**Table 3:** Vital clinical variables of subjects of the normal group and abnormal group stratified by BMI and WC standards after CRP.

Variables	All subjects	BMI standard		WC classified	
		Normal	Abnormal	Normal	Abnormal
Smoking (%)	18.3% (*n* = 61)	15.6% (*n* = 32)	20.7% (*n* = 29)	16.7% (*n* = 24)	18.9% (*n* = 37)
HR (bpm)	73.8 ± 11.9 (*n* = 61)	74.8 ± 14.1 (*n* = 32)	72.6 ± 8.8 (*n* = 29)	72.6 ± 15.1 (*n* = 24)	75.5 ± 9.4 (*n* = 37)
SBP (mmHg)	124.1 ± 13.9 (*n* = 61)	120.5 ± 13.9 (*n* = 32)	128.1 ± 13.0 (*n* = 29)	120.9 ± 14.7 (*n* = 24)	126.2 ± 13.2 (*n* = 37)
DBP (mmHg)	71.9 ± 9.3 (*n* = 61)	**68.4** ± **7.7** ^**^ (*n* = 32)	**75.7** ± **9.6** ^**^ (*n* = 29)	69.5 ± 7.7 (*n* = 24)	73.4 ± 10.0 (*n* = 37)
FBG (mmol/L)	6.4 ± 2.2 (*n* = 61)	6.6 ± 2.6 (*n* = 32)	6.2 ± 1.7 (*n* = 29)	6.4 ± 2.9 (*n* = 24)	6.4 ± 1.6 (*n* = 37)
TC (mg/dL)	155.7 ± 43.6 (*n* = 61)	158.6 ± 49.7 (*n* = 32)	152.5 ± 36.4 (*n* = 29)	146.4 ± 29.5 (*n* = 24)	161.7 ± 59.2 (*n* = 37)
TG (mg/dL)	71.6 ± 54.0 (*n* = 61)	68.3 ± 82.5 (*n* = 32)	75.2 ± 43.6 (*n* = 29)	58.1 ± 34.5 (*n* = 24)	80.3 ± 62.5 (*n* = 37)
LDL_C (mg/dL)	90.2 ± 31.2 (*n* = 61)	85.7 ± 24.2 (*n* = 32)	95.2 ± 37.3 (*n* = 29)	82.0 ± 18.5 (*n* = 24)	95.5 ± 36.5 (*n* = 37)
HDL_C (mg/dL)	43.7 ± 12.4 (*n* = 61)	45.0 ± 14.3 (*n* = 32)	42.3 ± 9.9 (*n* = 29)	**47.6** ± **15.3** ^*^ (*n* = 24)	**41.2** ± **9.5** ^*^ (*n* = 37)
UA (umol/L)	392.1 ± 100.5 (*n* = 61)	400.1 ± 107.0 (*n* = 32)	384.2 ± 93.9 (*n* = 29)	386.9 ± 103.1 (*n* = 24)	395.5 ± 100.0 (*n* = 37)
LVDd (mm)	50.9 ± 4.5 (*n* = 40)	50.9 ± 4.9 (*n* = 22)	50.8 ± 4.2 (*n* = 18)	51.3 ± 5.2 (*n* = 19)	50.5 ± 3.9 (*n* = 21)
IVSd (mm)	9.1 ± 1.5 (*n* = 40)	8.9 ± 1.5 (*n* = 22)	9.3 ± 1.5 (*n* = 18)	**8.4** ± **1.1** ^**^ (*n* = 19)	**9.7** ± **1.6** ^**^ (*n* = 21)
LVPWd (mm)	11.9 ± 2.0 (*n* = 40)	11.5 ± 2.0 (*n* = 22)	12.3 ± 2.0 (*n* = 18)	**11.0** ± **1.3** ^*^ (*n* = 19)	**12.6** ± **2.3** ^*^ (*n* = 21)
EF (%)	61.9 ± 8.7 (*n* = 40)	61.0 ± 8.7 (*n* = 22)	63.1 ± 8.8 (*n* = 18)	60.7 ± 9.6 (*n* = 19)	63 ± 7.9 (*n* = 21)
FS (%)	33.4 ± 6.1 (*n* = 40)	32.5 ± 5.7 (*n* = 22)	34.6 ± 6.5 (*n* = 18)	32.9 ± 6.6 (*n* = 19)	33.9 ± 5.7 (*n* = 21)
Treadmill exercise time (min)	8.16 ± 1.9 (*n* = 37)	**8.9** ± **2.1** ^*^ (*n* = 17)	**7.5** ± **1.6** ^*^ (*n* = 20)	8.8 ± 2.3 (*n* = 16)	7.7 ± 1.5 (*n* = 21)
Metabolic equivalents	10.2 ± 2.6 (*n* = 37)	11.0 ± 2.9 (*n* = 17)	9.6 ± 2.3 (*n* = 20)	11.1 ± 3.4 (*n* = 16)	9.5 ± 1.7 (*n* = 21)
Maximum net ST segment deviation (mm)	0.6 ± 0.8 (*n* = 37)	0.6 ± 0.7 (*n* = 17)	0.6 ± 0.9 (*n* = 20)	0.5 ± 1.0 (*n* = 16)	0.6 ± 0.7 (*n* = 21)
DTS	5.2 ± 5.1 (*n* = 37)	5.7 ± 4.7 (*n* = 17)	4.8 ± 5.4 (*n* = 20)	6.3 ± 5.7 (*n* = 16)	4.4 ± 4.5 (*n* = 21)

Notes: ^*^correlation is significant at the 0.05 level. ^**^Correlation is significant at the 0.01 level.

CRP: cardiac rehabilitation program; BMI: body mass index; WC: waist circumference; HR: heart rate; SBP: systolic blood pressure; DBP: diastolic blood pressure; FBG: fasting blood glucose; LDL_C: low density lipoprotein cholesterol; TC: total cholesterol; TG: triglycerides; HDL_C: high density lipoprotein cholesterol; UA: serum uric acid; LVDd: left ventricular and diastolic dimension; IVSd: interventricular septal thickness at diastole; LVPWd: left ventricular posterior wall at diastole; EF: ejection fraction; FS: fractional shortening; DTS: Duke treadmill score.

**Table 4:** The correlation between vital clinical variables and BMI or WC before and after CRP.

Variables	BMI				WC			
	Before CRP		After CRP		Before CRP		After CRP	
	*r*	*P*	*r*	*P*	*r*	*P*	*r*	*P*
SBP (mmHg)	0.287	**0.025** ^*^	0.461	**0.000** ^**^	0.205	0.113	0.371	**0.003** ^**^
DBP (mmHg)	0.279	**0.029** ^*^	0.542	**0.000** ^**^	0.184	0.157	0.309	**0.015** ^*^
FBG (mmol/L)	0.249	0.053	0.083	0.525	0.305	**0.017** ^**^	0.154	0.237
TC (mg/dL)	0.071	0.589	−0.043	0.740	0.174	0.179	0.159	0.220
TG (mg/dL)	0.177	0.173	0.091	0.487	0.027	0.838	0.109	0.402
LDL_C (mg/dL)	0.147	0.260	0.097	0.458	0.334	** 0.009** ^**^	0.204	0.115
HDL_C (mg/dL)	−0.235	0.068	−0.235	0.069	−0.256	**0.046** ^*^	−0.292	**0.022** ^*^
UA (umol/L)	0.149	0.251	0.093	0.476	0.369	** 0.003** ^**^	0.171	0.187
LVDd (mm)	−0.023	0.863	−0.095	0.559	0.017	0.899	0.044	0.786
IVSd (mm)	0.134	0.302	0.273	0.089	0.375	** 0.003** ^**^	0.451	**0.004** ^**^
LVPWd (mm)	0.179	0.166	0.271	0.091	0.309	**0.016** ^*^	0.468	** 0.002** ^**^
EF (%)	0.128	0.324	0.182	0.262	−0.101	0.439	0.036	0.824
FS (%)	0.072	0.582	0.225	0.163	−0.086	0.508	0.029	0.861
Treadmill exercise time (min)	−0.048	0.837	−0.269	0.107	−0.129	0.579	−0.164	0.333
Metabolic equivalents	−0.247	0.279	−0.267	0.110	−0.213	0.353	−0.211	0.210
Maximum net ST segment deviation (mm)	0.008	0.973	−0.022	0.897	−0.103	0.656	0.028	0.870
DTS	−0.123	0.595	−0.095	0.575	−0.168	0.466	−0.082	0.630

Notes: ^*^correlation is significant at the 0.05 level. ^**^Correlation is significant at the 0.01 level.

CRP: cardiac rehabilitation program; BMI: body mass index; WC: waist circumference; HR: heart rate; SBP: systolic blood pressure; DBP: diastolic blood pressure; FBG: fasting blood glucose; LDL_C: low density lipoprotein cholesterol; TC: total cholesterol; TG: triglycerides; HDL_C: high density lipoprotein cholesterol; UA: serum uric acid; LVDd: left ventricular and diastolic dimension; IVSd: interventricular septal thickness at diastole; LVPWd: left ventricular posterior wall at diastole; EF: ejection fraction; FS: fractional shortening; DTS: Duke treadmill score.

**Table 5:** The simple linear correlation between IVSd, LVPWd, and BMI/WC after CRP.

Dependent factor	Independent factor	*β*	Lower 95% CI	Upper 95% CI	*P*	*R* ^2^
IVSd	BMI After CRP	0.148	−0.023	0.319	0.089	0.074
	Constant	5.432	1.175	9.688	0.014	

IVSd	WC After CRP	0.08	0.028	0.131	**0.004** ^**^	0.203
	Constant	2.079	−2.502	6.659	0.364	

LVPWd	BMI After CRP	0.2	−0.034	0.433	0.091	0.219
	Constant	6.912	1.112	12.713	0.021	

LVPWd	WC After CRP	0.113	0.043	0.182	**0.002** ^**^	0.219
	Constant	1.945	−4.23	8.12	0.528	

Notes: ^*^the simple linear correlation is significant at the 0.05 level. ^**^Correlation is significant at the 0.01 level.

IVSd: interventricular septal thickness at diastole; LVPWd: left ventricular posterior wall at diastole; BMI: body mass index; WC: waist circumference; CRP: cardiac rehabilitation program; CI: confidence interval.

**Table 6:** The multiple linear correlation between IVSd, LVPWd, and WC after CRP.

Model	*β*	Lower 95% CI	Upper 95% CI	*P*	Adjusted *R* ^2^	*P*
Model 1: dependent factor: IVSd; independent factor: WC, BMI						
Constant	2.079	−2.502	6.659	0.364	0.182	**0.004** ^**^
WC after CRP (cm)	0.080	0.028	0.131	0.004		

Model 2: dependent factor: IVSd; independent factor: WC, age, smoking, hypertension, diabetes, TC, and HDL_C						
Constant	−2.847	−8.294	2.599	0.296	0.305	**0.001** ^**^
WC after CRP (cm)	0.098	0.046	0.150	0.000		
Age after CRP (y)	0.073	0.021	0.124	0.007		
Diabetes after CRP	−0.839	−1.841	0.163	0.098		

Model 3: dependent factor: LVPWd; independent factor: WC, BMI						
Constant	1.945	−4.230	8.120	0.528	0.198	**0.002** ^**^
WC after CRP (cm)	0.113	0.043	0.182	0.002		

Model 4: dependent factor: LVPWd; independent factor: WC, age, smoking, hypertension, diabetes, TC, and HDL_C						
Constant	−3.099	−10.571	4.373	0.406	0.269	**0.002** ^**^
WC after CRP (cm)	0.140	0.069	0.212	0.000		
Age after CRP (y)	0.063	−0.008	0.134	0.079		
Hypertension after CRP	−1.342	−2.647	−0.038	0.044		

Notes: ^*^the multiple linear correlation is significant at the 0.05 level. ^**^Correlation is significant at the 0.01 level.

IVSd: interventricular septal thickness at diastole; LVPWd: left ventricular posterior wall at diastole; WC: waist circumference; CRP: cardiac rehabilitation program; SBP: systolic blood pressure; DBP: diastolic blood pressure; LDL_C: low density lipoprotein cholesterol; CI: confidence interval.
